# Phylogenetic placement of the mysterious Malagasy genus *Ambrella* H. Perrier (Vandeae, Orchidaceae)

**DOI:** 10.3389/fpls.2025.1602122

**Published:** 2025-06-19

**Authors:** Sławomir Nowak, Marcin Górniak, Natalia Olędrzyńska, Przemysław Baranow, Dariusz L. Szlachetko

**Affiliations:** ^1^ Department of Plant Taxonomy and Nature Conservation, Faculty of Biology, The University of Gdansk, Gdansk, Poland; ^2^ Department of Evolutionary Genetics and Biosystematics, Faculty of Biology, The University of Gdansk, Gdansk, Poland

**Keywords:** Angraecinae, divergence time estimation, molecular taxonomy, Orchidaceae, phylogenetics

## Abstract

**Introduction:**

The monotypic genus *Ambrella* H. Perrier, endemic to Madagascar and traditionally placed within the subtribe Angraecinae, has long poses a challenge in terms of its phylogenetic classification.

**Methods:**

To clarify the evolutionary position of *Ambrella*, analyzed nuclear (ITS) and plastid (matK) markers applying maximum likelihood methods for phylogenetic inference and estimating divergence times using a Bayesian relaxed molecular clock model implemented in BEAST.

**Results:**

Our results show that *Ambrella* forms a strongly supported clade with the Malagasy genera *Cryptopus* Lindl., *Oeonia* Lindl., and *Neobathiea* Schltr. Despite the phylogenetic proximity, *Ambrella* remains morphologically distinct, particularly in terms of its floral architecture. According to molecular dating, the lineage leading to *Ambrella* diverged during the Late Miocene to Early Pliocene (ca. 8.8–3.3 Mya), a period of substantial ecological change in Madagascar.

**Discussion:**

The results of this study provide new insight into the evolutionary history of *Ambrella* and clarify its phylogenetic position within Vandeae. Despite forming a well-supported clade with other Malagasy genera such as *Cryptopus*, *Oeonia*, and *Neobathiea*, *Ambrella* maintains clear morphological distinctiveness, particularly in its floral architecture. This suggests that its most striking morphological features may have evolved independently.

## 1 Introduction

The systematics of orchids, as one of the largest families of flowering plants, has always been challenging. However, it is only in the past two decades, with the development of phylogenetic and now phylogenomic studies, that many changes have occurred in the way orchids are classified (Górniak et al., 2010; Chase et al., 2015; Pérez‐Escobar et al., 2021; Pérez-Escobar et al., 2024). Nevertheless, it seems that the phylogeny of this group is more complex than it might originally appear, and there are still many questions to be resolved. In this group of plants, as in no other, phenomena, such as hybridization, transfer of genetic material via other organisms, or incomplete lineage sorting, play an important role and complicate the pattern of phylogenetic relationships obtained through simple analyses of genetic markers.

One of the most diverse groups in Orchidaceae are species of the tribe Vandeae comprising mainly epiphytes from tropical regions. Recently, they have been classified under Epidendroideae, the largest subfamily among orchids (Chase et al., 2015; Freudenstein and Chase, 2015; Pérez-Escobar et al., 2024). The tribe includes four subtribes, Aeridinae, Adrorhizinae, Polystachyinae, and Angraecinae, with a relatively well-studied phylogeny (Russell et al., 2010; Mytnik-Ejsmont, 2011; Mytnik-Ejsmont et al., 2015; Simo-Droissart et al., 2018; Kim et al., 2020). However, there are still many gaps to be filled, including the need to place some taxa on the phylogenetic tree. One of these is the genus *Ambrella* H. Perrier, which has been included in the subtribe Angraecinae since it was first described (De La Bathie, 1934; Dressler, 1993; Szlachetko, 1995; Chase et al., 2015). The main objective of this work is to verify and check the placement of this unusual and somewhat mysterious genus in the angraecoid orchids based on molecular data.

**Figure 1:**
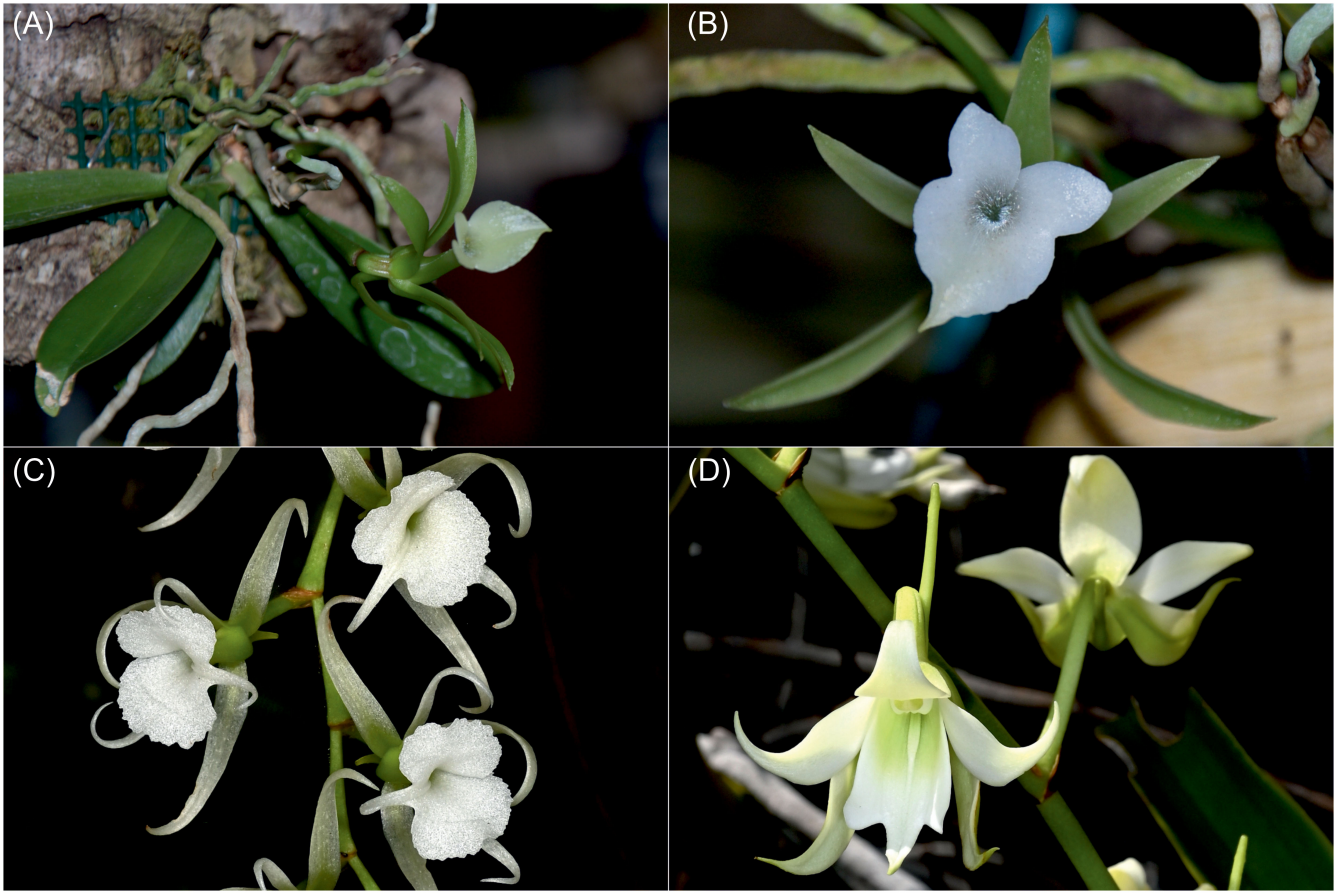
Plant with inflorescence **(A)** and frontal view of the flower **(B)** of *Ambrella longituba* H. Perrier, fragment of the inflorescence showing the flowers of *Oeoniella polystachys* (Thouars) Schltr. **(C)**, close-up of the flower of *Sobennikoffia humbertiana* H. Perrier **(D)** [photographs **(A, B)** taken by Stefano Pagnoni; **(C, D)** taken by Ron Parson].

*Ambrella* H. Perrier was described in 1934 in the Bulletin de la Société Botanique de France as a monotypic genus with the only representative being *A. longituba* H. Perrier, a species unique among angraecoid orchids in terms of flower morphology. Interestingly, in the protologue, the author compared his discovery with *Sobennikofia* Schltr. and *Oeoniella* Schltr., which are genera that differ significantly from *Ambrella* in many respects (**Figure 1**). Perrier De la Bathie (1934) emphasized its distinctness in the shape of the labellum and the arrangement of the reproductive organs, its much smaller size, broad, flat leaves, and greenish flowers when compared with the two genera mentioned. In the first description, *Ambrella* was characterized as unique and distinctly different from *Sobennikofia* and *Oeoniella* by having a long tubular labellum, hairy inside the tube, with a base that completely hides the column, and the median lobe of the rostellum thickened and exceeding the laterals, pollinia with tegulas, and separate, free viscidia (Perrier De la Bathie 1934).

*Ambrella longituba* was discovered in Madagascar, in Central Highlands, on the borders of the northern sector of the Eastern Highlands. The detailed location in the protologue states the river Makys, at approximately 800 m, on the northern side of the base of Mount Ambre, as an epiphyte on the branches of *Calliandra alternans* Benth., hanging over waterfalls or rapids. The species is known from only a few collections and is recognized as threatened (Bachman et al., 2024). As far as we know, the species is known in herbarium resources, only by the original material and collections made by *Dupuy s.n. (34)* from 1992 to 1995 in the same location, the vicinity of Joffreville, Mt. Ambre. All specimens are deposited in the Muséum National d’Histoire Naturelle in Paris, France. The species was also observed later (in 2022 by J. Hermans), and the population is estimated at fewer than 50 individuals and decreasing (Rabarimanarivo and Ramandimbisoa, 2024). Although the species is reported from Montagne d’Ambre National Park, it is facing the same threats as in neighboring areas, including forest degradation caused by illegal logging, illegal agricultural practices, fires, and grazing.

Considering the small population size in a limited distribution area and the threats, it is understandable that *Ambrella longituba* has been assessed as critically endangered (CR) according to the IUCN (Rabarimanarivo and Ramandimbisoa, 2024). Therefore, such steps as including the species in Madagascar’s list of protected species and its transfer to Appendix I of the Convention on International Trade in Endangered Species of Wild Fauna and Flora (CITES) are fully justified. In addition, active protection through propagation and introduction into the environment, combined with education and the involvement of the local community in efforts to address the problem of the disappearance of species, especially endemic and extremely rare ones, which are of enormous value to Madagascar, is worth considering.

## 2 Materials and methods

For phylogenetic reconstruction, we used 183 taxa that represent the subtribe Angraecinae, with *Polystachya* species as an outgroup. The accession numbers of sequences downloaded from GenBank (www.ncbi.nlm.nih.gov), as well as those newly generated for *Ambrella*, are provided in **Supplementary Table S1** (**Supplementary Materials**).

### 2.1 DNA isolation, amplification, and sequencing

Genomic DNA was extracted from approximately 20 mg of dried leaf tissue using the Genomic Mini Plant Kit (A&A Biotechnology, Poland) following the manufacturer’s instructions. Polymerase chain reaction (PCR) amplifications were performed in a total volume of 25 µl containing 1× buffer, 2 mM MgCl_2_, 0.2 mM dNTPs, 0.2 µM of each primer, 1.0 U of Color Perpetual Taq DNA polymerase (EURx Ltd. Poland), and DNA template (~30 ng). Amplified products were purified using the Wizard SV Gel and PCR Clean-UP System (Promega, USA).

The same primer pairs were used for both amplification and sequencing. Amplification of the ITS region (ITS1–5.8S–ITS2) was performed using primers 17SE and 26SE (Sun et al., 1994), while for the *mat*K region, primers −19F (Wilson et al., 2000) and 1326R (Cuénoud et al., 2002) were used. PCR thermal cycling conditions began with an initial denaturation at 94°C for 4 min, followed by 30 cycles of denaturation at 94°C for 45 s, annealing at 52°C for 45 s, and an extension step at 72°C for either 1 min (ITS) or 2 min (*mat*K) depending on the target region. Sequencing of the purified PCR products (~80 ng and 2.5 μM primer) was performed externally by Macrogen (Amsterdam, the Netherlands). Both DNA strands were sequenced to ensure accuracy in base calling. The sequences were edited using FinchTV v. 1.4.0 (Geospiza, Inc.), and the two complementary strands were assembled with AutoAssembler (ABI).

### 2.2 Phylogenetic analyses

DNA sequences were aligned using MAFFT v.7 (Katoh and Standley, 2013). Separate alignments were prepared for the ITS region (850 bp) and *mat*K gene (1,361 bp). For each dataset, the best-fit evolutionary model was estimated using IQ-TREE v.2. Due to the absence of significant topological conflicts, nuclear and plastid DNA data were combined into a single concatenated matrix (2,211 bp) for further analyses. Additional details about the datasets analyzed are presented in **Table 1**.

**Table 1:** Summary of character statistics for the analyzed datasets.

Character type	ITS	*mat*K	ITS x *mat*K
Total characters	850	1,361	2,211
Constant characters	311	733	1,247
Parsimony-informative characters	399	394	626

Phylogenetic reconstruction was performed using the maximum likelihood (ML) method in IQ-TREE v.2 (Minh et al., 2020). Clade support was assessed through a nonparametric bootstrap analysis with 1,000 replicates in the same software. Additionally, divergence time estimation within the studied group was conducted using BEAST v.1.8.4, executed on the CIPRES Science Gateway server (Miller et al., 2010). The analysis was run in two independent replicates, each consisting of 40 million generations. An uncorrelated relaxed molecular clock (lognormal) and the Yule model of speciation were applied. Two calibration points, following Givnish et al. (2015), were used: 27.34 million years ago (Mya) for the Polystachyinae node and 21.21 Mya for the ingroup. The results from both independent runs were assessed in Tracer v.1.6 to ensure convergence and subsequently combined in LogCombiner v.1.8.4 where 25% of the initial generations from each run were discarded as burn-in. The final maximum clade credibility (MCC) tree was generated using TreeAnnotator v.1.8.4.

## 3 Results

**Figure 2:**
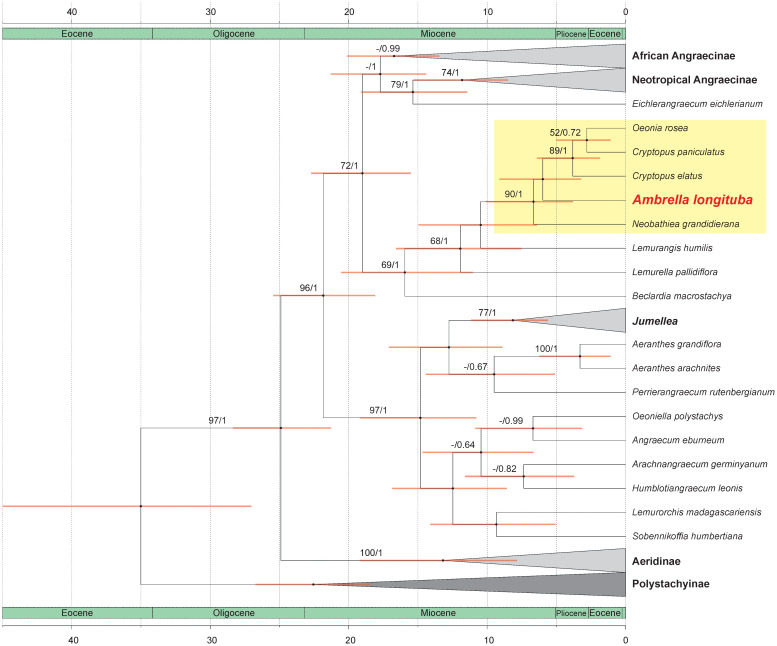
The maximum clade credibility tree presenting relationships within subtribe Angraecinae obtained from combined (nr ITS and plastid *mat*K) dataset using Bayesian inference. The divergence times (in Mya) are shown on the scale bars located at the top and bottom of the figure. Node support values are shown as PP/BS.

The results of our analyses are presented on a maximum clade credibility tree derived from a molecular clock analysis illustrating the divergence times of the studied lineages (**Figure 2**). Support values for individual clades are indicated above the branches as posterior probability (PP)/bootstrap support (BS).

The overall topology of the inferred phylogenetic tree is congruent with previous studies (Simo-Droissart et al., 2018; Farminhão et al., 2021) further supporting the robustness of the recovered relationships.

Our analyses strongly support the placement of the genus *Ambrella*, represented by *A. longituba*, within a well-supported clade (PP/BS = 90/1), grouping it with *Oeonia rosea* Ridl., *Cryptopus paniculatus* H. Perrier, *C. elatus* (Thouars) Lindl., and *Neobathiea grandidieriana* (Rchb.f.) Garay. This suggests a close evolutionary relationship among these taxa. The estimated divergence time for the most recent common ancestor (MRCA) of this group falls within the late Miocene to early Pliocene, approximately 8.83 to 3.31 Mya.

## 4 Discussion

### 4.1 Phylogenetic placement

**Figure 3:**
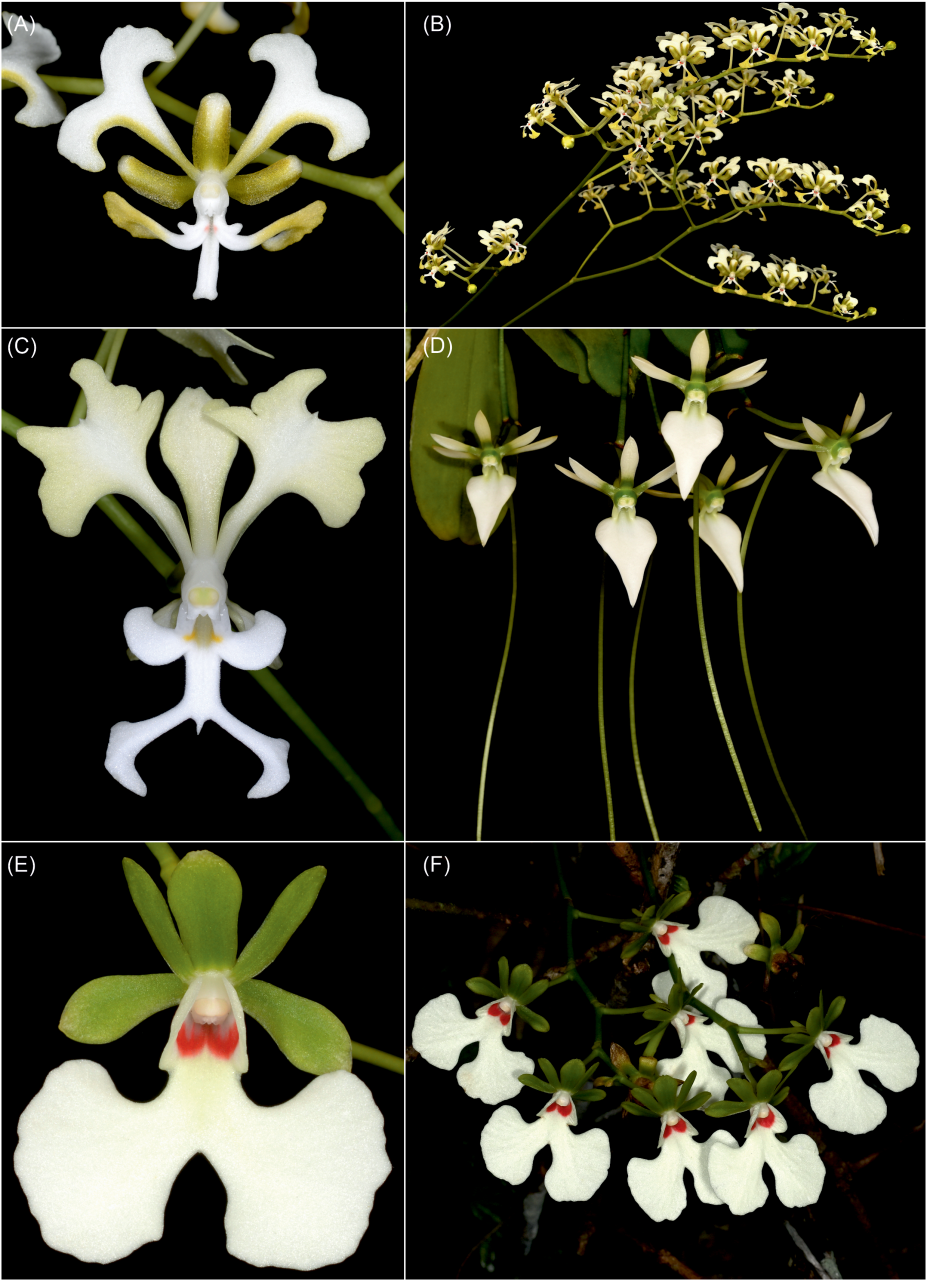
Single flower **(A)** and inflorescence **(B)** of *Cryptopus paniculatus* H. Perrier, close-up of the flower of *Cryptopus elatus* (Thouars) Lindl. **(C)**, single-flowered inflorescences of *Neobathiea grandidieriana* (Rchb.f) Garay **(D)**, single flower **(E)** and inflorescence **(F)** of *Oeonia rosea* Ridl. (photographs taken by Ron Parson).

Our investigations confirm the previous phylogenetic framework for Vandeae and especially for angraecoid orchids (Simo-Droissart et al., 2018). *Ambrella* is embedded in the clade along with the so-called vitsyangraecoides (Farminhão et al., 2021), which includes a few taxa found mainly in Madagascar and also in eastern Africa. The phylogenetic position of *Ambrella*, along with *Cryptopus* Lindl. and *Oeonia* Lindl., is strongly supported. Interestingly, both of these genera are very different from *Ambrella* (**Figure 3**). In both, we can observe elongated, ascending stems with loosely spaced leaves, at least several-flowered, elongated inflorescences, and wide-open flowers with a freely accessible gynostemium and spur entrance. The generative parts in representatives of both genera relate in structure to *Angraecum* Bory *s.str.*, i.e., the rostellum is dome shaped, with a weakly marked middle lobe. In contrast, in *Ambrella*, *Cryptopus*, and *Oeonia*, the pollinia are attached to two tegulae, at the base of which separate viscidia are present. The genus *Neobathiea* Schltr. is then sister to them, followed by the genus *Lemurella* Schltr., with the East African *Lemurangis humilis* (Summerh.) Szlach., Mytnik & Grochocka, a representative of the *Angraecum* complex (=*A. humile* Summerh.), and finally *Beclardia* A. Rich., which is sister to all of them. Of the genera mentioned, elongated stems with significantly distant leaves can be observed in *Neobathiea* (e.g., *N.* sp*atulata* H. Perrier), but already in *N. perrieri* (Schltr.) Schltr. or *N. hirtula* H. Perrier and *Lemurella*, the leaves have a similar form and distribution on the stem as in *Ambrella*. One could speculate that the primary feature in this evolutionary line, maintained in *Ambrella*, is the shortened stem and its relatively dense foliage. In *Ambrella* ancestors, there is a tendency to elongate the stem by making it ascending. This trait is undoubtedly an adaptation to habitat conditions. In this clade, a clear differentiation in the morphology of the floral parts is observed, while the structure of the generative parts is maintained. In all these genera, with the exception of *Beclardia*, the short, dome-shaped rostellum, with a weakly marked central patch, is present, and the pollinia are attached to two tegulae, with an independent viscidium on each. In *B. macrostachya* (Thouars) A. Rich., the rostellum is strongly elongated, and the equally elongated tegula is attached to a single viscidium. Both pollinia are attached to the apical, disc-shaped extended part of the tegula.

Such a resolution of phylogeny is consistent with previous studies (Simo-Droissart et al., 2018; Farminhão et al., 2021). Interestingly, the vitsyangraecoides group is not most closely related to the other Malagasy angraecoids and is sister to the Afroneotropical clade. Thus, *Ambrella* is not closely related to the *Oeoniella* and *Sobennikoffia* compared in the protologue. In both genera, the stem is elongated, as are the leaves, and the inflorescences are multiflowered. The lip in *Oeoniella* is tubular, with a relatively narrow spur entrance and access to the gynostemium. The structure of the gynostemium in the two genera is different. In *Oeoniella*, the lateral lobes of the rostellum are elongate, the median lobe is strongly reduced, and the pollinia are attached to two tegulae that connect to a single viscidium. In *Sobennikoffia*, the lateral lobes of the rostellum are strongly reduced, with both pollinia attached to a common tegula, to which two independent viscidia are attached on the other side.

Despite being related to other taxa than assumed, *Ambrella* is morphologically a completely separate taxon. Vegetative traits in all Angraecinae are confusing, and taxonomic distinctiveness cannot usually be recognized, but flower architecture resolves this problem. The inflorescence of *Ambrella longituba*, with one to three flowers, does not resemble the multifloral inflorescence of *Cryptopus* and *Oeonia* or uniflowered inflorescence in *Neobathiea.* Finally, the tubular labellum, with hairs inside and very short spur observed in *Ambrella*, is completely different from the spurless flowers of *Cryptopus* and *Oeonia*, which have a flat labellum, at most slightly tubular at the base, through fusion with the gynostemium. Moreover, the *Neobathiea* has a very long, downward-hanging spur, which is typical of many angraecoid orchids.

### 4.2 Divergence time

*Ambrella longituba* falls within a clade of taxa that separated in the Early Miocene (ca. 8.8–3.3 Mya), and diversification within it continued up to the Pliocene. The Miocene period is a time of significant changes in the vegetation of Africa (Couvreur et al., 2021). Due to the cooling of the climate in the middle Miocene, many forest formations experienced decline and division especially on the continent, but it could also be the reason for the lack of diversification or extinction in Madagascar (Buerki et al., 2013). The estimated divergence time for the most recent common ancestor of *Ambrella* and sister genera falls within the Late Miocene to Early Pliocene, approximately 8.83 to 3.31 Mya. This is a time of ongoing changes in the vegetation of Madagascar and the development and expansion of humid forests (Buerki et al., 2013), which provide the main habitat for *Ambrella* and closely related taxa, promoting their cladogenesis.

### 4.3 Pollination possibilities

Representatives of the Angraecinae are fairly well known for their mechanisms of pollination by long-proboscis insects (Lepidoptera), which is related to the presence in many species of a long spur containing nectar (Micheneau et al., 2014; Netz and Renner, 2017). However, other groups have also been observed as effective pollinators in the subtribe such as Diptera and Hymenoptera (Singer and Cocucci, 1999) for *Campylocentrum aromaticum* Barb.Rodr. or even Orthoptera (Micheneau et al., 2010) for *Hadrangis cadetii* (Bosser) Szlach., Mytnik & Grochocka (=*Angraecum cadetii* Bosser).

Nothing is known, so far, about pollination in *Ambrella longituba*. According to the note of Perrier left under the protologue (1924), it may be associated with a dependence on a narrow group of potential pollinators. The insect that inserts its head into a small window at the base of the lip to pump nectar from the spur can only exit through a window covered by two pollen-connecting stipes. The narrowness of this window must allow pollination of this flower only by a few very exceptional insects. The role of the hairs inside the tubular labellum remains unclear. Such a feature is often an adaptation to zoogamy, and in many plants, such a closed pathway to the reproductive organs is evidence of trap flowers (Case and Bradford, 2009; Matallana-Puerto et al., 2024). The hairs may also be part of the attraction and food for pollinators (Davies et al., 2002).

Finally, self-pollination cannot be eliminated, which has been confirmed for other closely related taxa, such as *Cryptopus elatus*, *Oeonia rosea*, and *Beclardia macrostachya* (Thouars) A.Rich (Agnew, 1986; Jacquemyn et al., 2005). Only for *Neobathiea grandidieriana* was pollination by Lepidoptera observed (Nilsson et al., 1987), which is obviously related to the presence of a spur, which is also present in *Ambrella*, but certainly not in this form. Therefore, pollination in *Ambrella* remains an interesting question to investigate.

## 5 Conclusion

The results presented here confirm the previous position of the genus *Ambrella* within the subtribe Angraecinae. However, *Ambrella* is more closely related to genera such as *Cryptopus*, *Oeonia*, and *Neobathiea* than *Oeoniella* and *Sobennikoffia*, with which it has so far been compared. Therefore, the tubular lip that surrounds the gynostemium arose twice independently in *Ambrella* and *Oeoniella*. Both genera belong to two separate clades that diverged in the early Miocene. *Ambrella* falls within a group of several species-poor genera found mainly in Madagascar, sister to the Afro-Neotropical Angraeciinae, while *Oeoniella* is a part of a clade of Malagasy taxa that have reached a high diversity on the island.

## Data Availability

The datasets presented in this study can be found in online repositories. The names of the repository/repositories and accession number(s) can be found in the article/**Supplementary Material**.

## Supplementary material

Supplementary Table 1 Genbank accession numbers of the sequences used in this study. Newly generated sequences are marked with an asterisk (*).
